# Associations between coping styles, gender, their interaction and non-suicidal self-injury among middle school students in rural west China: A multicentre cross-sectional study

**DOI:** 10.3389/fpsyt.2022.861917

**Published:** 2022-08-09

**Authors:** Jinhui Zhou, Jiazhu Zhang, Yilin Huang, Jiayu Zhao, Yun Xiao, Shibei Zhang, Yanfeng Li, Ting Zhao, Jinyu Ma, Nanbing Ou, Shuyi Wang, Qing Ou, Jiaming Luo

**Affiliations:** ^1^School of Psychiatry, North Sichuan Medical College, Nanchong, China; ^2^Nanyang Centre for Public Administration, Nanyang Technological University, Singapore, Singapore; ^3^Nanchong Psychosomatic Hospital, Nanchong, China

**Keywords:** non-suicidal self-injury, coping styles, middle school students, gender, interactive effects

## Abstract

**Background:**

To investigate the association between coping styles, gender, their interactions and non-suicidal self-injurious (NSSI) behaviors among middle school students in rural western China under COVID-19.

**Methods:**

A multicentre cross-sectional study method was used to conduct an online survey of 8,361 students from 23 middle schools in the northern Sichuan region by clustering sampling, using the General Information Questionnaire, the Ottawa Self-Injury Inventory, and the Coping Style Scale for Middle School Students.

**Results:**

The past year prevalence of NSSI among middle school students in rural west China was 5.7%. The differences in scores between those with and without NSSI on all dimensions of coping styles were statistically significant (*p* < 0.001). Multivariate logistic regression analysis revealed that vocational high school (*OR* = 1.67), girls (*OR* = 2.5), single parent with divorced parents (*OR* = 1.89), remarriage with divorced parents (*OR* = 1.81), and tolerance (*OR* = 1.17), venting emotions (*OR* = 1.15) and fantasy/denial (*OR* = 1.07) in coping styles may increase the risk of NSSI among middle school students, while problem solving (*OR* = 0.9) and seeking social support (*OR* = 0.9) among coping styles may reduce the risk of NSSI among middle school students. The interaction results show that gender has a moderating role in the process of endurance, avoidance, venting of emotions, and fantasy/denial influencing non-suicidal self-injury in middle school students.

**Conclusion:**

There is an association between coping styles and self-injury among middle school students in rural areas in western China, with gender playing a moderating role. Active attention should be paid to students' coping styles and encouraging them to adopt positive coping styles as well as avoid negative coping styles, especially in the case of girls, which can help prevent self-injury.

## Introduction

Non-suicidal self-injury (NSSI) is the intentional infliction of injury to a physical surface without suicidal intent, which is not culturally accepted by society (1). There is often an overlap between NSSI and suicidal behavior, with patients who develop NSSI having a suicide rate 100 times higher than the general population in the following 12 months and continuing to have a high prevalence of suicide many years afterwards, with over 5% of patients eventually committing suicide within 9 years (2). A large number of studies have shown that adolescents are most susceptible to the disease (3). NSSI tends to start in adolescence and increase after 14 years of age (4, 5), and the prevalence of NSSI gradually decreases in youth (5). The results of a meta-analysis with Chinese middle school students as a sample show that the prevalence of NSSI among Chinese middle school students is 22.37% (6). NSSI has gradually become an important public health problem affecting the health of adolescents in China (6). In addition, China has a significant number of rural populations with many of them being adolescents, but medical resources and health care systems, two factors related to NSSI (7), are insufficient in these areas. Studies have also shown that adolescents in rural areas have poorer health literacy than urban ones (8). Therefore, it is crucial to study the status of NSSI among adolescents in rural China and its associated factors.

Coping style refers to an individual's changing cognitive and behavioral efforts to meet intrinsic and extrinsic needs that are beyond his or her ability to achieve (9). Coping styles can be divided into two categories, problem orientation and emotional orientation, based on their different functions and expressions. The former has a positive effect on the individual and consists of problem solving and seeking social support, while the latter has the opposite effect and consists of venting, avoidance, fantasy, denial, etc (10). It has been suggested that for adolescents, adopting a problem-oriented coping way, represented by problem solving, contributes to higher academic achievement (11) and higher life satisfaction (12), while an emotion-oriented coping style, represented by venting, was significantly associated with suicidal ideation in middle school students (13). More direct studies related to self-injury have shown that problem solving and help-seeking are protective factors for college students' NSSI, while avoidance and fantasy/denial are threatening factors (14). On the other hand, research has also shown that individuals tend to use self-injury as a way of coping with psychological pain and regulating emotions (15–17), which also indicates that adolescents with self-mutilation behaviors have no effective measures to cope with these emotions (18). As the association between coping styles and self-injury has been less frequently tested in secondary school student populations, one of the aims of this study was to examine the association between the two.

In addition, numerous studies have shown that self-injurious behaviors show significant differences by gender (19–22). There may be differences in the psychological mechanisms underlying the occurrence of non-suicidal self-injurious behaviors across genders (23–25). On the one hand, studies have found that women have higher prevalence of non-suicidal self-injury than men, and such gender differences are consistent for depression and anxiety (19). The reason may be that men and women typically regulate their emotions in different ways (26–28), and different coping styles mean different things to men and women. On the other hand, it has been suggested that boys and girls have different motivations for self-injury, with boys emphasizing social concerns and girls focusing on emotional expression (29). In a study on the relationship between alexithymia and self-injury, for example, alexithymia was positively associated with self-injury for girls and negatively associated with self-injury for boys (23, 24); while another study of the relationship between friendship quality and self-injury found that girls with low friendship quality were more likely to self-injure, while the opposite was true for boys (25). As there are differences in the motivations of boys and girls for self-injury, it can be assumed that these differences are also present in the relationship between coping styles and self-injury. Specifically, emotionally oriented coping styles may be more strongly associated with self-injury for girls.

In summary, this study hypothesized that problem-oriented coping would negatively associate with the occurrence of NSSI among middle school students, while emotion-oriented coping would positively associate with the occurrence of NSSI among middle school students. This study aims to find out the detection characteristics and risk factors of NSSI among middle school students in western rural areas of China, and to explore the relationship between middle school students' coping style, gender and their interaction and NSSI. The result can provide a theoretical basis for the prevention and control of NSSI in rural areas of Western China.

## Materials and methods

### Participants

The data of this study were collected from March 2020 to April 2020, when COVID-19 in China was severe, and strict epidemic prevention and quarantine measures were adopted across the country. At that time, all schools were closed and students participated in this study at home. Due to the epidemic, this survey was completed online. The data in this study are mainly collected from middle schools in Yingshan County, which is located in remote western China. This place is chosen because it is representative of rural western China: most of its families live in poverty and engage in agricultural labor, some in villages and some in nearby towns. First, the investigators entered the questionnaire into the Wenjuanxing platform (a professional online survey platform) to generate a QR code. After the subjects scanned the code, an informed consent form would appear on the first page and they can only sign the form to answer the questionnaire. Second, there are 67 middle schools in Yingshan County with 36,085 students and 20 townships. Taking the school as the unit, the stratified random sampling method was adopted. For the 17 smaller towns, one school was chosen each; for the larger towns, two schools were chosen from three towns. As a result, 23 schools were selected for this study. Students scan the QR code on their parents' WeChat app to fill in online; finally, 8,785 questionnaires were distributed in this survey, and 8,361 valid questionnaires were collected, with an effective recovery rate of 95.17%. This study was in accordance with the ethical principles, reviewed by the Ethics Committee of Nanchong Psychosomatic Hospital.

### Measures

#### Demographic characteristics

Self-made questionnaires were used to collect the socio-demographic information of middle school students including gender, *hukou* or household registration (urban indicates that their parents are non-agricultural workers and rural means the opposite), whether being left-behind children (Children under the age of 18 who stayed in the place of household registration for half a year or more and could not live with both parents while growing up because both parents or one of them work in other cities), whether being resident students (in China, middle school provides dormitories, and students can voluntarily choose whether to live in based on their family situation), age, whether being only children, parents' marital status (divorced, non-divorced), parents' educational level, parents' occupation, health conditions, etc.

#### The NSSI rating tool

The Ottawa Self-injury Inventory (OSI) (30) was developed by Nxion and Paula Cloutier. According to the DSM-5 criteria for determining NSSI, NSSI is defined as an act of deliberate self-harm of body or skin tissue that does not result in death. Self-injurious behaviors include self-cutting, burning, rubbing the skin, striking, and biting. This definition was used in conjunction with the relevant entries on the Ottawa Self-Injury Survey scale to determine whether the subjects had NSSI. The Ottawa Scale measures a lot of content, but this study's focus is on the prevalence of NSSI in the past year, so only items related to NSSI in the past year are included in this study. The subjects were asked “In the past year, have you performed self-cutting, burning, skin rubbing, beating, biting, or other related NSSI behaviors?” If the answer is yes, they need to report the number of NSSIs performed in a year. The OSI has good reliability and validity in Chinese adolescents (31).

#### Middle school students' coping styles

The coping styles scale developed by Shulin Chen was used (10). The scale contains seven coping dimensions, including problem solving (e.g., Make a problem-solving plan and implement it step by step), seeking social support (e.g., Ask classmates, family members or relatives for help to overcome difficulties), rationalization (e.g., I try to look at the problem from a different angle, and I see the positive side from the setbacks), tolerance (e.g., I bury unpleasant things in my heart), avoidance (e.g., Facing setbacks, I give up trying to get what I want), venting emotions (e.g., I get angry with people and things that cause difficulty), and fantasy/denial (e.g., In the face of difficulties, I often think “I wish it had never been true”). There are 36 items in total and the scale of “1 to 4” indicates the frequency of “never, occasionally, sometimes and often.” The α coefficients of each dimension of the coping scale in this survey were 0.90, 0.83, 0.82, 0.63, 0.72, 0.76 and 0.80. The English language translation of the scale can be found in the Supplementary materials to this article.

### Statistical analysis

Microsoft Excel was used to build the database, and logical checks and data screening were performed. SPSS 24.0 was used for the analysis of descriptive and inferential statistics. Specifically, whether demographic variables such as gender, whether being the only children, and whether being left-behind have differences in affecting NSSI were tested with χ^2^, and the effect size Cramer's V value was reported. To test the relationship between coping style and NSSI, an independent sample *t*-test was used, and the Cohen's d value of the effect size was reported. We considered 0.2 as a threshold for small effect, 0.5 for moderate effect, and 0.8 for large effect in case of Cohen's d, while the corresponding thresholds for Cramer's V were 0.1, 0.3 and 0.5, respectively. Logistic regression analysis was used to analyze the influencing factors of non-suicidal self-injury and the interaction of gender.

## Results

Among the 8,361 middle school students surveyed, 5,503 (65.81%) were junior high school students, 1,442 (17.24%) were high school students, 1,416 (16.93%) were vocational high school students; 4,397 (52.58%) were boys and 3,964 were girls (47.41%). The mean age was (14.62 ± 1.91) years.

### Past year prevalence of NSSI in school students and its characteristics

Among the 8,361 middle school students surveyed, 476 had experienced non-suicidal self-injurious behavior and the prevalence of NSSI was 5.7%. There was an association between NSSI among middle school students and school category, gender, whether they were left behind, whether they lived at school and parents' marital status (all *p* < 0.05), and no association was seen with the place of residence, whether they were only children, father's education and mother's education (all *p* > 0.05). The prevalence of NSSI was higher for middle school students attending general high schools than for junior high school students, and higher for those attending vocational high schools. The prevalence of NSSI was higher in girls, left-behind children, and resident students. In terms of marital status, divorced and remarried families had the highest prevalence of NSSI, followed by single-parent families, and children in families without divorce (two parents) had the lowest prevalence of NSSI of the three. Cramer's V ≤ 0.1, and the effect size is at a low level, as detailed in Table 1.

**Table 1:** Descriptive analysis of the prevalence of NSSI in the past year.

**Group**		**N**	**Prevalence of NSSI (%)**	***χ^2^***	***P* value**	***Cramer's V***
Education qualification	Junior high school	5,503	258 (4.7)	46.5	*P* <0.001	0.08
	Senior high school	1,442	85 (5.9)			
	Vocational high school	1,416	133 (9.4)			
Gender	Boys	4,397	158 (3.6)	76.1	*P* <0.001	0.10
	Girls	3,964	318 (8.0)			
Residence place	Rural	3,821	237 (6.2)	3.4	0.065	0.02
	Urban	4,540	239 (5.3)			
Only child or not	Yes	1,177	71 (6.0)	0.2	0.588	0.005
	No	7,184	405 (5.6)			
left-behind or not	Yes	3,422	219 (6.4)	5.3	0.02	0.03
	No	4,399	257 (5.2)			
Resident or not	Yes	2,616	184 (7.0)	12.7	*P* <0.001	0.04
	No	5,747	269 (5.1)			
Parents'marital status	Parents	7,068	343 (4.9)	60.1	*P* <0.001	0.08
	Divorced single parent	770	78 (10.1)			
	Divorced and remarriage	523	55 (10.5)			
Father's educational qualification	Lower than junior high school	6,939	409 (5.9)	3.7	0.156	0.02
	High school/junior college	1,083	54 (5.0)			
	Tertiary and above	339	13 (3.8)			
Mother's educational qualification	Lower than junior high school	7,357	426 (5.8)	1.6	0.445	0.01
	High school/junior college	778	41 (5.3)			
	Tertiary and above	226	9 (4.0)			

### Comparison of coping styles between groups with and without NSSI

Comparing the scores of different dimensions of coping styles between the group with NSSI and the group without NSSI, the ANOVA results showed that problem solving, seeking social support, rationalization, tolerance, avoidance, venting emotions, and fantasy/denial dimension scores were all statistically significant differences between the NSSI and non-NSSI groups, as detailed in Table 2.

**Table 2:** Comparison of students' scores on the dimensions of coping styles in the no NSSI and NSSI groups ($\bar{x}\pm$s).

**Responding in all dimensions**	**Non-NSSI** **(*N* = 7,885)**	**NSSI** **(*N* = 476)**	***T* value**	***P*-value**	***Cohen's d***
Problem solving	19.4 ± 5.4	16.9 ± 4.5	11.14	*P* <0.001	0.5
Seeking social support	17.3 ± 5.03	15.8 ± 4.5	6.86	*P* <0.001	0.31
Rationalization	13.3 ± 3.8	12.2 ± 3.3	7.05	*P* <0.001	0.31
Tolerance	8.8 ± 2.8	10.06 ± 2.6	−9.23	*P* <0.001	−0.47
Avoidance	7.4 ± 2.7	8.4 ± 2.6	−8.63	*P* <0.001	−0.38
Venting of emotions	7.5 ± 2.8	9.04 ± 3.09	−10.53	*P* <0.001	−0.52
Fantasy/denial	9.1 ± 3.6	11.1 ± 3.5	−11.58	*P* <0.001	−0.56

For coping styles, the scores of different dimensions were compared between groups with NSSI and without NSSI. The independent sample *t*-test results showed that the scores of problems solving, seeking social support, positive rationalization, tolerance, avoidance, venting emotions, and fantasy/denial have a statistically significant difference between the NSSI group and the non-NSSI group. The effect size of problem solving, venting emotions, and fantasy/denial reached a medium level (|Cohen's d|>0.5), while the four dimensions of seeking social support, positive rationalization, tolerance, and avoidance are at a low level (|Cohen's d|>0.2), as shown in Table 2.

### Logistic regression analysis of risk factors of NSSI

A multivariate logistic regression analysis was conducted using NSSI as the dependent variable (yes = 1, no = 0) and the statistically significant variables corresponding to middle school students in the above one-way comparative analysis as independent variables. The variables were coded, where school category was referenced to junior high school, gender was referenced to male students, whether left behind was referenced to no, whether living in school was referenced to no, and parental marital status was referenced to both parents. The results showed that after controlling for other influencing factors, girls were more likely to engage in NSSI behavior than boys, vocational high school students were more likely to engage in NSSI behavior than junior high school students, living in a divorced and remarried family was more likely to engage in NSSI behavior than in a two-parent family, and children from divorced single-parent families were more likely to engage in NSSI behavior than the former two; problem solving was adopted and seeking social support problem-oriented coping reduced the occurrence of NSSI, while adopting tolerance, venting emotions and fantasy/denial middle school students directed emotion coping increased the occurrence of NSSI, as detailed in Table 3.

**Table 3:** Multivariate logistic regression analysis of middle school students' NSSI behavior (*N* = 8,361).

**Independent variable**		***B* value**	***Wald* value**	***OR* (95%CI)**	***P*-value**
Education qualification	Junior high school		15.6		*P* <0.001
	Senior high school	0.17	1.3	1.19 (0.8~1.6)	0.253
	Vocational high school	0.51	15.3	1.68 (1.2~2.1)	*P* <0.001
Gender		0.91	77.4	2.5 (2.0~3.0)	*P* <0.001
left-behind or not		−0.03	0.1	0.96 (0.7~1.1)	0.711
Resident or not		0.03	0.06	1.03 (0.8~1.3)	0.800
Parents' marital status	Parents		28.4		*P* <0.001
	Divorced single parent	0.63	19.9	1.89 (1.4~2.5)	*P* <0.001
	Divorced and remarriage	0.59	13.06	1.81 (1.3~2.4)	*P* <0.001
Problem solving		−0.09	23.3	0.91 (0.8~0.9)	*P* <0.001
Seeking social support		−0.1	33.7	0.9(0.8~0.9)	*P* <0.001
Rationalization		0.02	0.8	1.02 (0.9~1.07)	0.362
Tolerance		0.15	38.1	1.17 (1.1~1.2)	*P* <0.001
Avoidance		−0.006	0.06	0.99 (0.9~1.04)	0.795
Venting of emotions		0.13	35.9	1.15 (1.09~1.2)	*P* <0.001
Fantasy/denial		0.07	12.8	1.07 (1.03~1.1)	*P* <0.001
Constants		−3.78	275.1	0.02	

### Analysis of the interaction between factors of coping style and gender in relation to NSSI

The product terms of each factor of coping styles with gender were included separately on the basis of the baseline model. The results showed that, after controlling for a range of confounding factors such as parental marital status, being left behind, being a resident student, and grade in schools, the interaction terms for problem solving, seeking social support, and rationalization were not significant with gender, while the interaction terms for tolerance, avoidance, venting emotions, and fantasy/denial were significant with gender, as detailed in Table 4. Further simple subgroup analysis revealed that compared to boys, tolerance [*OR*_male_ = 1.08 (1.02 1.1) vs. *OR*_girls_ = 1.2 (1.1 1.2)], avoidance [*OR*_male_ = 1.07 (1.02 1.1) vs. *OR*_girls_ = 1.18 (1.1 1.2)], venting emotions [*OR*_male_=1.12 (1.07~1.1) vs. *OR*_girls_= 1.21(1.1~1.2)], and fantasy/denial [*OR*_male_=1.09 (1.05~1.1) vs. *OR*_girls_=1.16 (1.1~1.2)] four factors were more strongly associated with NSSI for girls.

**Table 4:** Regression analysis of the interaction of the factors of coping style and gender with NSSI.

**Models**	**Variables**	***B* value**	***Wald* value**	***OR value* (95%CI)**	***P*-value**
Model 1	Problem solving	−0.06	4.4	0.9 (0.8~0.9)	0.034
	Gender	0.97	8.7	2.6 (1.3~5.01)	0.003
	Problem solving*gender	−0.005	0.08	0.99 (0.9~1.03)	0.772
Model 2	Seeking social support	−0.08	5.6	0.92(0.8~0.9)	0.017
	Gender	0.6	3.5	1.9 (0.9~3.6)	0.058
	Seeking social support*gender	0.01	0.6	1.01 (0.9~1.05)	0.430
Model 3	Rationalization	−0.09	4.8	0.91 (0.8~0.9)	0.027
	Gender	0.6	4.08	1.96 (1.02~3.7)	0.043
	Rationalization*gender	0.01	0.4	1.01 (0.9~1.07)	0.496
Model 4	Tolerance	−0.02	0.2	0.97 (0.8~1.09)	0.632
	Gender	−0.12	0.1	0.88 (0.4~1.7)	0.729
	Tolerance*gender	0.11	9.3	1.11 (1.03~1.1)	0.002
Model 5	Avoidance	−0.02	0.1	0.98 (0.8~1.09)	0.687
	Gender	0.14	0.2	1.15 (0.6~2.05)	0.628
	Avoidance*gender	0.1	8.1	1.1 (1.03~1.1)	0.004
Model 6	Venting emotions	0.04	0.6	1.04 (0.9~1.1)	0.436
	Gender	0.27	0.8	1.3 (0.7~2.3)	0.353
	Venting emotions*gender	0.07	5.4	1.07 (1.01~1.1)	0.019
Model 7	Fantasy/denial	0.03	0.5	1.03 (0.9~1.1)	0.458
	Gender	0.27	0.9	1.3 (0.7~2.3)	0.340
	Fantasy/denial*gender	0.06	5.9	1.06 (1.01~1.1)	0.014

## Discussion

NSSI behavior is now widespread among middle school students worldwide (32) and is becoming an important public health problem among adolescents. In this study, a survey of 8,361 middle school students found that the past year's prevalence of NSSI was 5.7%, which is lower than in Italy (42%) (33), Canada (17%), Australia (6.2%) (34) and Germany (25.6%) (32), it is also lower than the prevalence of NSSI behavior among middle school students in China, which ranged from 12.2 to 22.37% (35, 36). There may be two reasons for this low detection rate. First, the data in this study were collected at the beginning of the COVID-19 outbreak, and the students had been at home for about 2–3 months under the supervision of their parents, resulting in less time for unsupervised self-harm. But this cannot fully explain the low rate as the data in this study were collected on the prevalence of NSSI over a 12-month period. Second, the tools used to detect NSSI can also cause the low rate. The commonly seen tools can be roughly divided into single-item detection and self-harm behavior checklist (37), and this study uses the former method, which, according to studies, has a far lower detection rate than the latter method (37, 38). This is also consistent with the results of the study in the Chinese region that also used similar method, in which the detection rate of students was 5.4% (39). The possible reason is that each item in the list acts as a recognition memory cue, while for a single item there are fewer cues and a lower sensitivity to NSSI detection, leading to differences in the two reports. For example, subjects will likely interpret the single-item assessment as asking if they are similar to self-mutilation populations, i.e., subjects may simply equate self-mutilation with wrists-cutting and other behaviors do not count. As a result, only self-perceived NSSI is measured. By contrast, behavioral inventory detection may define clearly, for example, “hitting the wall with a fist” as NSSI. But in single-item measurement, subjects who actually experience this behavior may not think it is NSSI. Therefore, a single assessment is beneficial to detect self-conceptual NSSI, while the behavioral checklist is beneficial to detect more NSSI patients in an objective concept. Researchers should choose different methods based on different purposes. Some literature also recommends using the two methods in combination to improve the detection accuracy (37). In short, the low detection rate of NSSI in this study is mainly due to the COVID-19 pandemic and the measurement method.

This study examined the association of many factors with NSSI, found that students attending vocational high schools had a significantly higher prevalence of NSSI than middle school students as well as general high schools, and that school category was also an independent risk factor for NSSI in the regression equation, which may be due to the lower self-efficacy and lower self-esteem of vocational high school students compared to their counterparts in general middle and high schools (40). The prevalence of NSSI by gender was statistically significant and also an independent risk factor for NSSI, which is also consistent with most previous studies (19–22). The difference in NSSI detection rates between those left-behind children and those children not left behind was statistically significant, which may be related to the lack of care and emotional support from parents (41). The difference in prevalence of NSSI between those who lived in school and those who did not was statistically significant, with the prevalence of NSSI among students who lived in school being significantly higher than that of non-resident students, which may be because of the fact that students who lived in school had more opportunities to learn and imitate NSSI behaviors from their peers. The difference in prevalence of NSSI by parental marital status was statistically significant and was also an independent risk factor in the regression equation, with students in divorced single-parent and divorced-remarried families having higher prevalence of NSSI behaviors compared to two-parent families (42), which also suggests that the marital status of parents has a significant impact on the psychological development of middle school students.

Through independent samples *t*-test, this study found that, in all dimensions of coping style, the differences between the presence and absence of NSSI were statistically significant. Specifically, the NSSI group scored significantly lower than the non-NSSI group on problem-oriented coping styles such as problem solving, seeking social support, and positive rationalization, while the NSSI group scored significantly higher than the non-NSSI group on emotion-oriented coping styles such as tolerance, avoidance, venting, fantasy/denial. The NSSI group scored significantly higher than the non-NSSI group for emotion-oriented coping styles such as tolerance, avoidance, venting emotions, and fantasy/denial. Further multivariate logistic regression analysis revealed that the five dimensions, i.e., problem solving, social support seeking, tolerance, venting and fantasy/denial, were significantly associated with NSSI. Problem solving and seeking social support are protective factors for NSSI, and previous research has also shown that adolescents with NSSI are less able to solve problems compared to those without NSSI (43), which probably links to the fact that problem-solving coping involves confronting the problem head-on, may resulting in a higher sense of self-efficacy and satisfaction of competence needs. On the other hand, seeking social support is beneficial for gaining relational and emotional support, as well as for meeting relational needs, and research has found that adolescents' sense of social support is effective in preventing the onset of NSSI (44, 45). The emotion-focused coping way usually leads to negative results, such as suicide ideation (12), which is in line with previous research (13, 46), this study also found that emotionally oriented coping styles such as tolerance, venting, and fantasy/denial were risk factors for NSSI, which may be explained by the fact that NSSI often appears as a result of failed emotion regulation (47). In particular, the adoption of emotion suppression may increase distressing emotions, thereby increasing the risk of NSSI in adolescents (44, 48).

The results of the interaction analysis showed that the four factors of tolerance, avoidance, venting of emotions, and fantasy/denial represented by emotional orientation in secondary school students' coping styles interacted with gender on the occurrence of non-suicidal self-injurious behaviors among secondary school students. Specifically, the association between emotionally oriented coping styles and the risk of non-suicidal self-injurious behavior was stronger for girls, which may be due to the fact that girls have different motivations for engaging in non-suicidal self-injurious behavior compared to boys, with girls being more likely to report self-injury as a result of emotional regulation, whereas boys are more likely to report social motivations, such as gaining attention (29, 49). Previous research has also shown that women with alexithymia are more likely to engage in self-injurious behavior than men who also have alexithymia (23). Both current and previous studies suggest that there are gender differences in the psychological mechanisms by which self-injurious behavior occurs and that interventions for self-injury should be targeted.

The significance of this study is that it provides targeted recommendations for the prevention of NSSI in rural western China. First, we should pay more attention to the students of middle and vocational high schools, girls, and students with divorced parents in this region; secondly, we should guide students to adopt more positive coping styles to deal with problems, avoid using negative coping styles, and developing students' problem-oriented coping styles are crucial to defense against non-suicidal self-injury; finally, girls with emotionally oriented coping styles are more prone to non-suicidal self-injury, so in the process of intervening NSSI, attention should be paid to different intervention methods for students of different genders, and for girls, attention should be paid to their emotional expression. The strength of this study lies in the multi-center data and large sample, but the limitation is that it is only a cross-sectional study and cannot determine the causal relationship. Future research should conduct longitudinal follow-up studies to further explore the associated factors of NSSI among middle school students in rural areas of western China.

## Conclusion

To sum up, this study found that the past year prevalence of self-injury in rural areas in western China was 5.7%, and the related factors of self-injury were attending vocational high school and experiencing parental divorce. In coping style, the five dimensions of problem solving, seeking social support, tolerance, venting emotions, and fantasy/denial are independent predictors of non-suicidal self-injury. Gender plays a moderating role in the relationship between coping style and self-injury. In all dimensions of emotional orientation, the associations with self-injury were stronger for girls.

## Data availability statement

The datasets generated for this study are available on request to the corresponding author.

## Ethics statement

The studies involving human participants were reviewed and approved by Nanchong Psychosomatic Hospital. Written informed consent to participate in this study was provided by the participants' legal guardian/next of kin.

## Author contributions

JZho was involved in the study design, data analysis, and the composing of this manuscript. JL and JZhan provided the subject of this study, critically revised this manuscript, and wrote the data collection part of this manuscript. YH, JZhao, YX, and SZ searched and reviewed the references. YL, TZ, JM, and NO collected the data. SW and QO modified this manuscript. All authors contributed to this manuscript and approved the submitted version.

## Funding

This work was financially supported by the Funding Project of the Bureau of Science and Technology and Intellectual Property of Nanchong City (No. 20YFZJ0101).

## Conflict of interest

The authors declare that the research was conducted in the absence of any commercial or financial relationships that could be construed as a potential conflict of interest.

## Publisher's note

All claims expressed in this article are solely those of the authors and do not necessarily represent those of their affiliated organizations, or those of the publisher, the editors and the reviewers. Any product that may be evaluated in this article, or claim that may be made by its manufacturer, is not guaranteed or endorsed by the publisher.
