# Supplementary material for: Associations between coping styles, gender, their interaction and non-suicidal self-injury among middle school students in rural west China: A multicentre cross-sectional study
Source: Front Psychiatry. 2022 Aug 9;13:861917. doi: 10.3389/fpsyt.2022.861917 (PMC9395723; doi:10.3389/fpsyt.2022.861917)
Supplement: Supplementary file 1 [file Table_1.DOCX]

Coping styles scale

1 Use my own or someone else's experience to deal with difficulties

2 Ask someone with experience or someone who has a similar experience

3 I see some good things from the bad things that have happened

4 When encountering difficulties, I think that "taking a step back enables me to see a wider world"

5 I have the fantasy that I can use superhuman skills to overcome difficulties

6 Strive to change the status quo and make things better

7 Try to get "what to do" advice from someone

8 I try to look at the problem from a different angle, and I see the positive side from the setbacks

9 Facing setbacks, I give up trying to get what I want

10 I love to do some unrealistic fantasies to eliminate worries

11 Think hard about “how can I best solve problems”

12 Ask classmates, family members or relatives for help to overcome difficulties

13 I learned something helpful from a difficult experience

14 Acknowledging that I can't handle the problem at hand, I’d give up trying

15 In the face of difficulties, I often think "it's just not true"

16 Work hard to find solutions to problems

17 Tell others about my troubles

18 I see difficulties and setbacks as part of my life experience

19 In the face of setbacks, I often admonish myself that "patience is the best"

20 Get upset when encountering difficulties and setbacks, and such this emotion is expressed

21 I often hope that when I wake up, the problem has been solved

22 Make a problem-solving plan and implement it step by step

23 I want emotional support from family, relatives or friends

24 I think "Life experience is suffering"

25 I bury unpleasant things in my heart

26 I get angry with people and things that cause difficulty

27 Refuse to believe bad things have happened

2 8 Learn from past failures to solve the difficulties ahead

29 Expect sympathy and understanding from others

30 I only have limited ability, so I can only endure some unpleasant things

31 When I encounter setbacks, I give up or lower my goals

32 If I can't solve the problem, I can be very distressed and vent to my family and friends

33 Do things that will solve the problem step by step

34 Discuss solutions to problems with classmates, friends or family

35 Take a wait-and-see attitude towards problems and let them develop

36 If I have unpleasant emotions, I will find some way to vent

Coping dimensions

**Problem solving:**

1 Use my own or someone else's experience to deal with difficulties

6 Strive to change the status quo and make things better

11 Think hard about “how can I best solve problems”

16 Work hard to find solutions to problems

22 Make a problem-solving plan and implement it step by step

28 Learn from past failures to solve the difficulties ahead

33 Do things that will solve the problem step by step

**Seek social support:**

2Ask someone with experience or someone who has a similar experience

7 Try to get "what to do" advice from someone

12 Ask classmates, family members or relatives for help to overcome difficulties

17 Tell others about my troubles

23 Want emotional support from family, relatives or friends

29 Expect sympathy and understanding from others

34 Discuss solutions to problems with classmates, friends or family

**Positive rationalization:**

3 I see some good things from the bad things that have happened

8 I try to look at the problem from a different angle, and I see the positive side from the setbacks

13 I learned something helpful from a difficult experience

1 8 I see difficulties and setbacks as part of my life experience

24 I think "Life experience is suffering"

**Tolerance:**

19 In the face of setbacks, I often admonish myself that "patience is the best"

25 I bury unpleasant things in my heart

30 I only have limited ability, so I can only endure some unpleasant things

35 I take a wait-and-see attitude towards problems and let them develop

**Avoidance:**

4 When encountering difficulties, I think that "taking a step back enables me to see a wider world"

9 Facing setbacks, I give up trying to get what I want

14 Acknowledging that I can't handle the problem at hand, I’d give up trying

31 When I encounter setbacks, I give up or lower my goals

**Venting emotions:**

20 Get upset when encountering difficulties and setbacks, and such this emotion is expressed

26 I get angry with people and things that cause difficulty

32 If I can't solve the problem, I can be very distressed and vent to my family and friends

36 If I have unpleasant emotions, I will find some way to vent

**Fantasy/Denial:**

5 I have the fantasy that I can use superhuman skills to overcome difficulties

10 I love to do some unrealistic fantasies to eliminate worries

15 In the face of difficulties, I often think "I wish it had never been true"

21 I often hope that when I wake up, the problem has been solved

27 I refuse to believe bad things have happened
